# Segurança, Eficácia e Protocolo de Dose de Metoprolol para Redução de Frequência Cardíaca em Pacientes Pediátricos Externos que Passaram por Angiografia Cardíaca por TC

**DOI:** 10.36660/abc.20190892

**Published:** 2021-01-27

**Authors:** Mariana de Oliveira Nunes, Dawn R. Witt, Susan A. Casey, Larissa I. Stanberry, David J. Caye, Bradford J.Chu, B. Jana Lindberg, John R. Lesser, B. Kelly Han

**Affiliations:** 1 Minneapolis Heart Institute and Foundation Minnesota EUA Minneapolis Heart Institute and Foundation, Minnesota - EUA; 2 Children’s Minnesota Minnesota EUA Children’s Minnesota, Minneapolis, Minnesota – EUA

**Keywords:** Cardiopatias Congênitas, Frequência Cardíaca, Metoprolol, Antagonistas Adrenérgicos, Tomografia Computadorizada, Vasos Coronários

## Abstract

**Fundamento:**

Qualidade de imagem e dose de radiação são otimizadas com uma frequência cardíaca (FC) lenta e estável na realização de imagens de artérias coronárias durante a angiografia cardíaca por tomografia computadorizada (CCTA, do inglês *cardiac computed tomography angiography* ) A segurança, a eficácia e o protocolo para a redução da FC com medicamento betabloqueador ainda não foi bem descrita em uma população de pacientes pediátricos.

**Objetivo:**

Oferecer um protocolo de dose de metoprolol eficiente a ser usado em pacientes pediátricos externos durante a CCTA.

**Métodos:**

Realizamos uma revisão retrospectiva de todos os pacientes pediátricos externos que receberam o metoprolol durante a CCTA. As características demográficas e clínicas foram resumidas e a redução média em FC foi estimada utilizando-se um modelo de regressão linear multivariada. As imagens foram avaliadas em uma escala de 1 a 4 (1= ideal).

**Resultados:**

Um total de 78 pacientes externos passaram a uma CCTA com o uso de metoprolol. A média de idade foi de 13 anos, a média de peso foi de 46 kg, e 36 pacientes (46%) eram do sexo masculino. As doses médias de metoprolol foram 1,5 (IQR 1,1; 1,8) mg/kg, e 0,4 (IQR 0,2; 0,7) mg/kg para administrações orais e intravenosas, respectivamente. O produto dose-comprimento por exame foi de 57 (IQR 30, 119) mGy*cm. A redução média da FC foi 19 (IQR 12, 26) batimentos por minuto, ou 23%. Não foram relatadas complicações ou eventos adversos.

**Conclusão:**

O uso de metoprolol num cenário de pacientes pediátricos externos para redução da FC antes de uma CCTA é seguro e eficiente. Pode-se reproduzir um protocolo de dose de metoprolol quando for necessário atingir uma FC mais lenta, garantindo tempos de aquisição mais rápidos, imagens mais claras e redução na exposição à radiação nessa população. (Arq Bras Cardiol. 2021; 116(1):100-105)

## Introdução

A angiografia cardíaca por tomografia computadorizada (CCTA) é o padrão de imagem para avalição não invasiva das artérias coronárias em pacientes de todas as idades.^1 , 2^ Para otimizar a qualidade da imagem e a dose de radiação, prefere-se ter uma FC mais baixa e estável.^3 , 4^ A redução da FC pode ser alcançada pelo uso de medicamento betabloqueador. Exames de imagem de artérias coronárias em crianças apresentam desafios específicos, devido ao tamanho dos vasos e FC mais alta em repouso. A principal modalidade diagnóstica para imagem coronária em pacientes com doença cardíaca congênita (DCC) historicamente tem sido a cateterização cardíaca, que exige anestesia, acesso vascular central, administração de contraste, e exposição significativa à radiação. A imagem cardíaca por ressonância magnética é útil para a imagem coronária, mas tem valor limitado em pacientes mais jovens.^5^ A CCTA demonstrou ser uma forma de diagnóstico em bebês e crianças de todas as idades, utilizando tomógrafos de última geração com resolução espaço-temporal adequada.^6 - 8^

Técnicas de otimização de dose de radiação diminuíram significativamente a exposição em comparação com tecnologias de tomografia anteriores. A imagem coronária pode ser adquirida em um único batimento ou em alguns batimentos cardíacos durante uma apnéia em pacientes de todas as idades.^9^ A FC mais baixa e estável permite o uso de uma janela estreita de aquisição de exposição à radiação, durante a sístole ou a diástole, dependendo da FC. A segurança e a eficácia da redução da FC com medicamento betabloqueador já está bem descrita para imagem coronária na população adulta,^10 - 12^ mas é rara para pacientes pediátricos.^6 , 13^ O objetivo desse estudo retrospectivo foi avaliar a segurança e a eficácia, e definir o protocolo de dosagem de metoprolol para redução da FC em uma população de pacientes pediátricos externos que passaram por CCTA.

## Métodos

### Pacientes

Foram incluídos no estudo pacientes entre 6 e 18 anos de idade que compareceram como pacientes externos e receberam o metoprolol antes da CCTA, no período de 1º de janeiro de 2007 a 31 de dezembro de 2016. Pacientes que fizeram a TC por indicação que não fosse uma DCC, que fizeram a CCTA sem o medicamento metoprolol, que foram encaminhados para a imagem coronária como pacientes internos, ou que eram pacientes externos, mas fizeram a tomografia anestesiados para garantir a cooperação com a suspensão da respiração foram excluídos do estudo. A FC de linha de base foi medida na apresentação do paciente externo no centro de imagens antes da administração do metoprolol e novamente durante a tomografia. A dose de metoprolol, a qualidade da imagem, e quaisquer eventos adversos foram documentados. O estudo foi aprovado pelo Comitê de Análise Institucional.

### Plataforma do Tomógrafo e Sequência de Varredura, e Preparação do Paciente

A CCTA foi realizada utilizando-se um tomógrafo de fonte dupla de primeira, segunda ou terceira geração (Somatom Definition Flash, Siemens Healthcare, Forchheim, Alemanha) com tempo de rotação do tubo = 280 ms, resolução temporal = 66-83 ms, e colimação =2×128×0,6 mm. Uma CCTA sincronizada ao eletrocardiograma (ECG), prospectiva e com alto pitch (3,4) foi realizada utilizando-se modulação de corrente de tubo online automatizada para uma FC lenta e estável < 55 batimentos por minuto (bpm), com tomógrafo de fonte dupla de segunda ou terceira geração. Nos casos de FC mais alta ou irregularidade significativa de FC, apesar do betabloqueador, foi realizado uma aquisição retrospectiva sincronizada ao ECG (Mindose) ou uma aquisição sequencial foi feita com a janela de aquisição ajustada conforme a FC. Tipicamente, foi utilizada uma janela de aquisição mais larga que incluiu a sístole para FC acima de 60 bpm. Quando se suspeitou de lesões coronárias nos pacientes com sintomas de isquemia ou doença de Kawasaki, foi usado uma aquisição retrospectiva sincronizada ao ECG (Mindose) ou uma aquisição sequencial, independente da FC, para permitir a avaliação de mais de um único conjunto de dados. Para todos os pacientes, a voltagem do tubo foi ajustado a um valor mais baixo baseado no uso do software automatizado Care kV (Siemens, Forchheim) ou na avaliação clínica. Em 2011, a voltagem do tubo a 70 kV foi disponibilizado com uma melhoria do tomógrafo. Os exames foram reconstruídos usando o algoritmo de reconstrução iterativa de segunda geração da Siemens, o Safire, na potência 3. Em 2014, uma terceira geração de algoritmos de reconstrução iterativa, o Admire, passou a ser usado, também na potência 3. A dose de contraste foi injetada na velocidade apropriada para a idade e o calibre intravenoso. O contraste foi injetado através de bomba injetora utilizando um cateter de calibre 20-24, com base no tamanho do paciente.

### Avaliação da Qualidade da Imagem

As imagens foram analisadas retrospectivamente por dois leitores especialistas (KH e BC), qualitativamente, conforme uma escala de quatro pontos: 1=totalmente aceitável com visualização ideal de todos os alvos anatômicos; 2=boa qualidade de imagem com visualização diagnóstica de todos os alvos anatômicos; 3=qualidade marginal da imagem, com visualização da maioria dos alvos anatômicos; e 4=má qualidade da imagem, não diagnóstica para a avaliação dos alvos anatômicos. Quaisquer discrepâncias na pontuação da qualidade da imagem foram revisadas novamente por KH e resolvidas. Os alvos anatômicos foram definidos como a capacidade de definição clara dos óstios coronários e a sua origem da grande artéria, definição clara do trajeto da coronária incluindo o relacionamento com as grandes artérias e o esterno, e a capacidade de identificar a anatomia da coronária distal para determinar a dominância coronária. Todas as tomografias com uma pontuação > 1 foram consideradas abaixo do ideal. Para essas tomografias, foi determinado o motivo para classificação da imagem como subótima.

### Estimativa da Dose de Radiação

O produto dose-comprimento do procedimento, em mGy*cm foi utilizada para estimar a dose de radiação. Um cilindro de 32 cm foi usado para estimativas de produto dose-comprimento em todos os pacientes, independentemente do tamanho. A dose de radiação é relatada como sendo o produto dose-comprimento do exame.

### Protocolo de Administração de Metoprolol

Um protocolo de metoprolol padrão foi usado para todos os pacientes incluídos neste estudo. As crianças passaram por triagem para determinar as contraindicações do uso de betabloqueador, incluindo estenose aórtica grave, hipertensão pulmonar de moderada a grave, ou disfunção sistólica ventricular esquerda ou direita grave. Pacientes com histórico de qualquer uma dessas entidades clínicas não receberam o betabloqueador. Quando a FC basal era < 60 bpm, o metoprolol não foi administrado. Quando a FC basal ficava entre 60 e 70 bpm, 1 mg/kg de metoprolol, até a dose máxima de 100 mg, foi administrado. Quando a FC basal era > 70 bpm, 2 mg/kg de metoprolol, até a dose máxima de 100 mg, foram administrados. Quando a FC permanecia acima de 70 bpm uma hora após a dose oral, 0,2 mg/kg de metoprolol intravenoso era administrada até a dose máxima de 1 mg/kg, para pacientes < 20 kg, ou dose máxima de 20 mg de dose intravenosa total, eram administrados para pacientes com peso acima de 20 kg. Se a FC no tomógrafo estiver > 70 bpm quando a FC basal tiver sido aceitável, o metoprolol intravenoso só foi administrado de acordo com as diretrizes acima.^14^

### Métodos Estatísticos

Os dados demográficos e clínicos do paciente foram resumidos usando contagens (%) para variáveis categóricas, médias ± desvio padrão para variáveis contínuas simetricamente distribuídas, e medias (faixas interquartis) para variáveis contínuas distorcidas. A mudança da FC após a administração do betabloqueador foi estimada utilizando-se um modelo de regressão linear multivariada, com a diferença em FC como variável de resposta, e idade, sexo, o produto dose-comprimento e a dose de metoprolol como covariáveis. As premissas do modelo foram definidas usando-se análise residual e o teste Shapiro-Wilk de normalidade. As estimativas do modelo, seus intervalos de confiança (CI) de 95%, e os p-valores foram relatados. A análise foi realizada usando o R 3.5.2 em ambiente R-Studio 1.1.463.^14 , 15^Foi usado um nível de significância de 5%.

## Resultados

### Dados Demográficos dos Pacientes e Redução da Frequência Cardíaca

Identificamos 78 pacientes pediátricos que passaram por uma CCTA com o uso de metoprolol antes da aquisição da imagem no contexto de pacientes externos em nossa instituição entre janeiro de 2007 e dezembro de 2016. Destes, cinquenta e nove (75%) pacientes fizeram a tomografia para avaliar as coronárias, e 19 (25%) a fizeram para avaliar outro tipo de DCC. Os dados demográficos dos pacientes, a FC e o mecanismo de administração do betabloqueador estão descritos na Tabela 1 . A média de idade na tomografia foi 13,33 (IQR 10, 16) anos de idade, 36 pacientes (46%) eram do sexo masculino, e a média de peso foi de 46 (IQR 31, 61) kg. Um paciente recebeu nitroglicerina sem efeito adverso.

**Tabela 1:** – Dados demográficos dos pacientes e redução da frequência cardíaca

Variável	Todos	Apenas oral	Apenas IV	IV + Oral
Paciente, n (%)	78 (100)	51 (65)	4 (5)	23 (29)
Idade na tomografia, anos	13,0±3,3	13,1±3,4	10,2±4,6	13,3±2,7
Sexo masculino, n (%)	36 (46)	22 (43)	1 (25)	13 (57)
Peso, kg,*	46 (31, 61)	46 (29, 59)	32 (29, 58)	49 (36, 62)
FC inicial, bpm,*	78±15	74±11	91±26	87±16
FC na tomografia, bpm,*	60±11	56±9	73±16	66±11
Redução de FC, bpm,*	19±10	18±9	18±10	20±12
Redução relativa da FC, %,*	23 (16, 30)	24 (17, 30)	20 (17, 22)	23 (15, 33)

N: número; IV: intravenosa; kg: quilograma; FC: frequência cardíaca; bpm: batimentos por minuto. * Variáveis contínua são relatadas como ± desvios padrão médios ou como faixas médias e interquartis (IQR, 25°, 75° percentis) se distorcidas. Variáveis categóricas resumidas por contagens (%).

No geral, a FC de linha de base foi de 77 (IQR 66, 90) bpm. A maioria dos pacientes, n = 51, (65%) recebeu apenas o metoprolol por via oral, e quatro pacientes (5%) receberam apenas o metoprolol intravenoso. Os demais pacientes receberam uma combinação de metoprolol via oral e intravenoso, n = 23 (29%). Após a administração do metoprolol, houve uma redução de 23% na FC basal que corresponde a 19 bpm, IQR (12-26). A partir da análise multivariada, a redução estimada na FC foi 20 bpm, com 95% IC (17,24) (Apêndice 1).

### Administração de Metoprolol

A dose de metoprolol depende do peso do paciente, conforme definido no Protocolo de administração de metoprolol, descrito anteriormente. Para os pacientes que pesavam ≤ 50 kg, a dose média de metoprolol oral ou intravenoso foi de 1,6 mg/kg (IQR 1,3, 1,9) e 0,6 mg/kg (IQR 0,3, 0,8), respectivamente. Para os pacientes que pesavam acima de 50 kg, a dose média de metoprolol oral ou intravenoso foi de 1,4 mg/kg (IQR 1,0, 1,6) e 0,3 mg/kg (IQR 0,1, 0,5), respectivamente. ( Tabela 2 ) As doses e quantidades administradas na prática eram consistentes com as especificadas em nosso protocolo clínico.^14^

**Tabela 2:** – Protocolo de Betabloqueadores - Dose e entrega por peso

Variável	Todos	Peso ≤ 50 kg	Peso > 50 kg
Dose oral, mg/kg (n=74*)	1.5±0.5	1.7±0.5	1.3±0.4
Dose IV, mg/kg (n=27*)	0.5±0.3	0.6±0.3	0.4±0.2
Quantidade oral, mg	50 (50, 100)	50 (50, 75)	100 (62, 100)
Quantidade IV, mg	20 (15, 28)	20 (13, 23)	20 (18, 35)

MG: miligramas; kg: quilogramas; IV: intravenosa; *inclui os que receberam IV e oral Variáveis contínua são relatadas como ± desvios padrão médios ou como faixas médias e interquartis (IQR, 25°, 75° percentis) se distorcidas.

### Dose de Radiação e Detalhes da Imagem

A Tabela 3 apresenta a dose de radiação da tomografia e detalhes da imagem. A média do produto dose-comprimento do exame foi de 57 (IQR 30, 119) mGy*cm. A pontuação média da qualidade da imagem foi de 1,2. Das 78 tomografias, 11 (14%) ficaram abaixo da qualidade ideal, com 10 casos pontuados como “2” devido ao contraste ruim e/ou ruído, e um caso foi classificado como “3”, devido a movimento do paciente. A representação das sequências de imagem foi uniforme, com aproximadamente um terço dos pacientes incluídos em cada tipo de sequência. Não foram relatadas complicações durante os procedimentos de imagem por CCTA ou depois do procedimento até o momento da alta do ambiente de paciente externo.^15^

**Tabela 3:** – Dose de radiação da tomografia e detalhes da imagem

DLP, mGy*cm*	57 (30, 119)
Qualidade da imagem da tomografia*	
1, n(%) (%)	67 (86)
2, n(%) (%)	10 (13)
3, n(%) (%)	1 (1)
4, n(%) (%)	0 (0)
**Sequência de imagem**	
ECG anatômico-prospectivo disparado com alta frequência (Flash), n (%)	26 (33)
ECG anatômico-prospectivo disparado (Prospectivo), n (%)	25 (32)
ECG retrospectivo-funcional (espiral), n (%)	27 (35)

DLP: produto dose-comprimento (do inglês dose-length product); mGy*cm. * variáveis categóricas resumidas por contagens (%); variáveis contínuas relatadas como médias e faixas de interquartil (IQR, 25º 75º percentis). Descrição da qualidade da imagem 1=totalmente aceitável com visualização ideal de todos os alvos anatômicos; 2=boa qualidade de imagem com visualização diagnóstica de todos os alvos anatômicos; 3=qualidade marginal da imagem, com visualização da maioria dos alvos anatômico; e 4=má qualidade da imagem, não diagnóstica para a avaliação de alvos anatômicos

## Discussão

Em pacientes adultos submetidos a CCTA, o uso de betabloqueador com controle da FC adequada demonstrou que melhora a qualidade da imagem.^16^ A pré-medicação por via oral demonstrou ser eficiente na população adulta, embora a variação da eficácia seja afetada pela dosagem.^16^ Já está bem documentado que os riscos de exposição repetida a anestesia e radiação ionizante devem ser evitadas para todos os pacientes de DCC.^17 - 21^Portanto, uma FC mais lenta permite o uso de eletrocardiograma prospectivo de disparo, que demonstrou reduzir significativamente a dose para angiografia coronária.^22^ Em nossa experiência, o uso de metoprolol intravenoso uma hora após a dose oral não teve efeito adicional na redução da FC. Portanto, interrompemos a administração de metoprolol intravenoso após a dose oral em nossa população de pacientes pediátricos a partir de 2013. Deve-se notar que três pacientes receberam o metoprolol IV depois de 2013 devido a FC elevada durante a aquisição do topograma por sofrerem de ansiedade. A redução da FC nas populações pediátricas pode ser alcançada com segurança e eficiência com um protocolo de administração de metoprolol padronizado, para pacientes submetidos a avaliação de CCTA no contexto de pacientes externos. Com triagem cuidadosa para contraindicações, não encontramos complicações ou efeitos colaterais com o uso de betabloqueadores em pacientes pediátricos.

### Limitações

Esse breve relatório está limitado a achados relacionados a FC e o uso de metoprolol, e não tem um grupo de comparação. Os autores concordam que um desenho prospectivo teria sido robusto. Entretanto, essa foi uma revisão retrospectiva que analisou nossa prática clínica. Também é importante mencionar que os leitores deste estudo não fizeram a leitura cega, o que poderia introduzir um viés.

## Conclusão

Um protocolo de dose de metoprolol na população pediátrica externa com DCC, antes da aquisição da CCTA, demonstrou ser segura e eficaz na redução de frequência cardíaca em pacientes entre 6 e 18 anos de idade. Um controle de frequência cardíaca adequado na população, utilizando o metoprolol, pode oferecer imagens mais claras devido à redução da movimentação e artefato, e garantindo tempo de aquisição menores e, portanto, associados à redução da exposição à radiação.
