# Supplementary material for: Segurança, Eficácia e Protocolo de Dose de Metoprolol para Redução de Frequência Cardíaca em Pacientes Pediátricos Externos que Passaram por Angiografia Cardíaca por TC
Source: Arq Bras Cardiol. 2021 Jan 27;116(1):100–5. [Article in Portuguese] doi: 10.36660/abc.20190892 (PMC8159495; doi:10.36660/abc.20190892)
Supplement: Translation

# Translation: Safety, Efficacy, and Dose Protocol of Metoprolol for Heart Rate Reduction in Pediatric Outpatients Undergoing Cardiac CT Angiography

**Authors:** Mariana de Oliveira Nunes, Dawn R. Witt, Susan A. Casey, Larissa I. Stanberry, David J. Caye, Bradford J.Chu, B. Jana Lindberg, John R. Lesser, B. Kelly Han

**Affiliations:** 1 USA Minneapolis Heart Institute and Foundation, Minnesota - USA; 2 USA Children’s Minnesota, Minneapolis, Minnesota – USA

**Keywords:** Heart Defects, Congenital, Heart Rate, Metoprolol, Adrenergic, Antagonists, Computed, Tomography, Coronary Vessels

## Abstract

**Background:**

Image quality and radiation dose are optimized with a slow, steady heart rate (HR) when imaging the coronary arteries during cardiac computed tomography angiography (CCTA). The safety, efficacy, and protocol for HR reduction with beta blocker medication is not well described in a pediatric patient population.

**Objective:**

Provide a safe and efficient metoprolol dose protocol to be used in pediatric outpatients undergoing CCTA.

**Methods:**

We conducted a retrospective review of all pediatric outpatients who received metoprolol during CCTA. Demographic and clinical characteristics were summarized and the average reduction in HR was estimated using a multivariate linear regression model. Images were evaluated on a 1-4 scale (1= optimal).

**Results:**

Seventy-eight pediatric outpatients underwent a CCTA scan with the use of metoprolol. The median age was 13 years, median weight of 46 kg, and 36 (46%) were male. The median doses of metoprolol were 1.5 (IQR 1.1, 1.8) mg/kg and 0.4 (IQR 0.2, 0.7) mg/kg for oral and intravenous administrations, respectively. Procedural dose-length product was 57 (IQR 30, 119) mGy*cm. The average reduction in HR was 19 (IQR 12, 26) beats per minute, or 23%. No complications or adverse events were reported.

**Conclusion:**

Use of metoprolol in a pediatric outpatient setting for HR reduction prior to CCTA is safe and effective. A metoprolol dose protocol can be reproduced when a slower HR is needed, ensuring faster acquisition times, clear images, and associated reduction in radiation exposure in this population. (Arq Bras Cardiol. 2021; 116(1):100-105)

## Introduction

Cardiac computed tomography angiography (CCTA) is the imaging standard for non-invasive assessment of coronary arteries in patients of all ages.^1 , 2^To optimize image quality and radiation dose, a slower and steady HR is preferred.^3 , 4^A reduction in HR can be achieved by using beta blocker medication. Imaging coronary arteries in children presents unique challenges due to smaller vessel size and higher resting HR. The main diagnostic modality for coronary imaging in congenital heart disease (CHD) patients has historically been cardiac catheterization, requiring anesthesia, central vascular access, contrast administration, and significant radiation exposure. Cardiac magnetic resonance imaging is useful for coronary imaging in older children but has limited value in the youngest patients.^5^CCTA has been shown to be diagnostic in infants and children of all ages using latest generation scanner technology with appropriate spatial and temporal resolution.^6 - 8^

Radiation dose optimization techniques have significantly decreased radiation exposure as compared to earlier scanner technology. Coronary imaging can be reproducibly acquired in a single heart beat or in several heart beats during a single breath hold sequence in patients of all ages.^9^A slower, steady HR allows for the use of a narrow acquisition window for radiation exposure during systole or diastole depending on HR. The safety and efficacy of HR reduction with beta blocker medication is well described for coronary imaging in the adult population,^10 - 12^but is scarce in the pediatric setting.^6 , 13^The purpose of this retrospective study was to evaluate the safety and efficacy, and define a dosage protocol of metoprolol for HR reduction in an outpatient population of pediatric patients who underwent CCTA.

## Methods

### Patients

Patients between 6 and 18 years of age were included if they presented as an outpatient and received metoprolol prior to CCTA from January 1, 2007 to December 31, 2016. Patients were excluded if they underwent a CT scan for a non-CHD indication, underwent CCTA without metoprolol medication, or were referred for coronary imaging from the inpatient setting or presented as an outpatient but were scanned under anesthesia for cooperation with suspended respiration. The baseline HR was measured at presentation to the outpatient imaging center prior to administration of metoprolol medication and again during the scan. Metoprolol dose, image quality, and any adverse events were documented. The study was approved by the Institutional Review Board.

### Scanner Platform, Scan Sequence, and Patient Preparation

CCTA were performed using a first, second, or third -generation dual-source CT scanner (Somatom Definition Flash, Siemens Healthcare, Forchheim, Germany) with gantry rotation time = 280ms, temporal resolution=66-83ms, and collimation=2×128×0.6 mm. A prospectively electrocardiogram-triggered high-pitch (3.4) scan was performed using automated online tube current modulation for slow and steady HR < 55 beats per minute (bpm) with the second or third generation dual source scanner. For higher HR or significant HR irregularity despite beta blocker, a retrospective electrocardiogram gated (Mindose) or sequential scan was done with the acquisition window adjusted for HR. Typically, a wider acquisition window that included systole was used for HR above 60 bpm. When coronary lesions were suspected in patients with symptoms of ischemia or Kawasaki disease, a retrospective electrocardiogram-gated (Mindose) or a sequential scan was used regardless of HR to allow evaluation of more than a single dataset. The tube potential was adjusted for all patients to a lower value based on the use of the automated software Care kV (Siemens, Forchheim) or on clinical judgement. In 2011, a 70 kV peak tube potential became available with a scanner upgrade. Scans were reconstructed using the Siemens second-generation iterative reconstruction algorithm, Safire, at a strength of 3. In 2014, a third- generation iterative reconstruction algorithm, Admire, began to be used, also with a strength of 3. Contrast dose was injected at the rate appropriate for age and intravenous gauge. Contrast was power-injected using a 20-24-gauge catheter based on patient size.

### Image Quality Assessment

Images were retrospectively reviewed by two expert readers (KH and BC) qualitatively on a four-point scale: 1=fully acceptable with optimal visualization of all anatomical targets; 2=good image quality with diagnostic visualization of all anatomical targets; 3=marginal image quality with diagnostic visualization of most anatomical targets; and 4=poor image quality, non-diagnostic for evaluation of anatomical targets. Any discrepancies in the scoring of image quality were re-reviewed by KH and reconciled. Anatomic targets were defined as the ability to see clear definition of coronary ostia and origin from the great artery; clear definition of coronary course, including relationship to great arteries and sternum; and the ability to identify distal coronary vessel anatomy to determine coronary dominance. All scans with a score >1 were considered suboptimal. For these scans, the reason for the suboptimal image quality was determined.

### Radiation Dose Estimation

Procedural dose length product in mGy*cm was used to estimate the radiation dose. A 32 cm phantom was used for dose length product estimates in all patients regardless of size. Radiation dose is reported as scan dose length product.

### Metoprolol Administration Protocol

A standard metoprolol protocol was used for all patients included in this study. Children were screened for contraindications to beta blockade including severe aortic stenosis, moderate to severe pulmonary hypertension, or severe left or right ventricular systolic dysfunction. Patients with a history of any of these clinical entities were not given beta blocker medication. If the baseline HR was < 60 bpm, metoprolol was not administered. If the baseline HR was between 60-70 bpm, 1 mg/kg metoprolol to maximum oral dose of 100 mg was administered. If the baseline HR was > 70 bpm, 2 mg/kg metoprolol to maximum oral dose of 100 mg was administered. If the HR remained over 70 bpm one hour after oral dose, 0.2 mg/kg intravenous metoprolol was given to a maximal dose of 1 mg/kg for patients < 20 kg, or maximum of 20 mg total intravenous dose was given for those over 20 kg. If the HR in the scanner is > 70 bpm when baseline HR was acceptable, intravenous metoprolol only was given according to guidelines above.^14^

### Statistical Methods

Patient demographic and clinical data were summarized using counts (%) for categorical variables, means ± standard deviations for symmetrically-distributed continuous variables, and medians (interquartile ranges) for skewed continuous variables. The change in HR following beta blocker administration was estimated using a multivariate linear regression model with difference in HR as a response variable and age, gender, dose length product, and metoprolol dose as covariates. Model assumptions were verified using residual analysis and the Shapiro-Wilk test for normality. The model estimates, their 95% confidence intervals (CI), and p-values are reported. The analysis was performed using R 3.5.2 in R-Studio 1.1.463 environment.^14 , 15^The significance level of 5% was used.

## Results

### Patient Demographics and Heart Rate Reduction

We identified 78 pediatric patients who underwent a CCTA scan with the use of metoprolol prior to image acquisition in the outpatient setting at our institution between January 2007 and December 2016. Fifty nine (75%) patients had the CCTA scan to assess coronaries and 19 (25%) had the study to assess another type of CHD. Patient demographics, HR, and beta blocker delivery mechanism are described in Table 1 . The median age at scan was 13.33 (IQR 10, 16) years, 36 (46%) were male and the median weight of 46 (IQR 31, 61) kg. One patient received nitroglycerin with no adverse event.

**Table 1:** – Patient demographics and heart rate reduction

Variable	All	Oral only	IV only	IV + Oral
Patient, n (%)	78 (100)	51 (65)	4 (5)	23 (29)
Age at scan, years	13.0±3.3	13.1±3.4	10.2±4.6	13.3±2.7
Male, n (%)	36 (46)	22 (43)	1 (25)	13 (57)
Weight, kg,*	46 (31, 61)	46 (29, 59)	32 (29, 58)	49 (36, 62)
HR initial, bpm,*	78±15	74±11	91±26	87±16
HR at scan, bpm,*	60±11	56±9	73±16	66±11
HR reduction, bpm,*	19±10	18±9	18±10	20±12
Relative reduction in HR, %,*	23 (16, 30)	24 (17, 30)	20 (17, 22)	23 (15, 33)

N: number; IV: intravenous; kg: kilogram; HR: heart rate; bpm: beats per minute. * Continuous variables are reported as means ± standard deviations or as medians and interquartile (IQR, 25^*th,*^75^*th*^percentile) ranges if skewed. Categorical variables summarized by counts (%).

Overall, the baseline HR was 77 (IQR 66, 90) bpm. The majority of patients, n = 51, (65%) received oral metoprolol only and four patients (5%) received intravenous metoprolol only. The remainder of the patients received a combination of oral and intravenous metoprolol n = 23 (29%). Following the metoprolol administration, there was a 23% reduction in baseline HR that corresponds to 19 bpm, IQR (12-26). From the multivariate analysis, the estimated reduction in HR was 20 bpm 95% CI (17, 24) (Appendix 1).

### Metoprolol Administration

Metoprolol dose is dependent on patient’s weight as outlined in the Metoprolol Administration Protocol previously described. For those weighing less than ≤ 50 kg, the median oral and intravenous metoprolol dose was 1.6 mg/kg (IQR 1.3, 1.9) and 0.6 mg/kg (IQR 0.3, 0.8), respectively. For patients weighing over 50 kg, the median oral and intravenous metoprolol dose was 1.4 (IQR 1.0, 1.6) and 0.3 (IQR 0.1, 0.5) mg/kg, respectively ( Table 2 ). The doses and amounts administered in practice are consistent with those specified in our clinical protocol.^14^

**Table 2:** – Beta Blocker Protocol- Dose and Delivery by Weight

Variable	All	Weight ≤ 50 kg	Weight > 50 kg
Dose oral, mg/kg (n=74*)	1.5±0.5	1.7±0.5	1.3±0.4
Dose IV, mg/kg (n=27*)	0.5±0.3	0.6±0.3	0.4±0.2
Amount oral, mg	50 (50, 100)	50 (50, 75)	100 (62, 100)
Amount IV, mg	20 (15, 28)	20 (13, 23)	20 (18, 35)

Mg: milligrams; kg: kilogram; IV: intravenous; *includes those that received both IV and oral. Continuous variables are reported as means ± standard deviations or as medians and interquartile (IQR, 25th, 75th percentile) ranges if skewed.

### Radiation Dose and Imaging Details

Table 3 provides scan radiation dose and imaging details. The median procedural dose-length product was 57 (IQR 30, 119) mGy*cm. The mean image quality score was 1.2. Out of 78 scans, 11 (14%) were of suboptimal quality with 10 cases scored as a “2” due to poor contrast and/or noise and one case ranked “3” due to patient motion. The representation of the imaging sequences was uniform, with approximately a third of patients included in each sequence type. No complications were reported during CCTA imaging procedures or after the procedure until the time of discharge from the outpatient setting.^15^

**Table 3:** – Scan Radiation Dose and Imaging Details

DLP, mGy*cm*	57 (30, 119)
Scan image quality*	
1, n (%)	67 (86)
2, n (%)	10 (13)
3, n (%)	1 (1)
4, n (%)	0 (0)
**Imaging Sequence**	
Anatomic-Prospective ECG triggered with high pitch (Flash), n (%)	26 (33)
Anatomic-Prospective ECG triggered (Prospective), n (%)	25 (32)
Functional-Retrospective ECG gated (Spiral), n (%)	27 (35)

DLP: dose-length product; mGy*cm. *categorical variables summarized by counts (%); continuous variables reported as median and interquartile (IQR, 25^*th,*^75^*th*^percentile) ranges. Description of image quality: 1=fully acceptable with optimal visualization of all anatomical targets; 2=good image quality with diagnostic visualization of all anatomical targets; 3=marginal image quality with diagnostic visualization of most anatomical targets; and 4=poor image quality, non-diagnostic for evaluation of anatomical targets

## Discussion

In adult patients undergoing CCTA, beta blocker use with adequate HR control has been shown to improve image quality.^16^Oral pre-medication has been shown to be effective in the adult population, although variation in efficacy is affected by dosing.^16^It is well documented that risks of repeated exposure to anesthesia and ionizing radiation for all CHD patients should be avoided.^17 - 21^Therefore, a slower HR allows for the use of prospective electrocardiogram triggering, which has been shown to significantly reduce radiation dose for coronary angiography.^22^In our experience, intravenous metoprolol after an oral dose did not have an additional effect on reducing HR. Therefore, we have discontinued administration of intravenous metoprolol after oral dose in our pediatric patient population since 2013. Of note, three patients did receive IV metoprolol after 2013 due to elevated HR during topogram acquisition due to anxiety. HR reduction in pediatric populations can be safely and effectively achieved with a standardized metoprolol delivery protocol for patients undergoing CCTA assessment in the outpatient setting. With careful screening for contraindications, we found no complications or side effects with the use of beta blockers in pediatric patients.

### Limitations

This report is limited to findings regarding HR and metoprolol use and does not have a comparison group. The authors agree that a prospective design would have been more robust; however, this was a retrospective review that analyzed our clinical practice. Furthermore, the readers for this study were not blinded, which could introduce bias.

## Conclusion

A metoprolol dose protocol in the outpatient pediatric population with CHD before the acquisition of cardiac CTA showed safety and efficacy in heart rate reduction in patients between 6 and 18 years of age. An adequate heart rate control in pediatric population with metoprolol can provide clearer images due to reduction in motion and artifact, ensure faster acquisition times, and reduce radiation exposure.
